# Light-induced negative differential resistance in graphene/Si-quantum-dot tunneling diodes

**DOI:** 10.1038/srep30669

**Published:** 2016-07-28

**Authors:** Kyeong Won Lee, Chan Wook Jang, Dong Hee Shin, Jong Min Kim, Soo Seok Kang, Dae Hun Lee, Sung Kim, Suk-Ho Choi, Euyheon Hwang

**Affiliations:** 1 Department of Applied Physics and Institute of Natural Sciences, Kyung Hee University, Yongin 446-701, Korea; 2 SKKU Advanced Institute of Nanotechnology, Sungkyunkwan University, Suwon 440-746, Korea

## Abstract

One of the interesing tunneling phenomena is negative differential resistance (NDR), the basic principle of resonant-tunneling diodes. NDR has been utilized in various semiconductor devices such as frequency multipliers, oscillators, relfection amplifiers, logic switches, and memories. The NDR in graphene has been also reported theoretically as well as experimentally, but should be further studied to fully understand its mechanism, useful for practical device applications. Especially, there has been no observation about light-induced NDR (LNDR) in graphene-related structures despite very few reports on the LNDR in GaAs-based heterostructures. Here, we report first observation of LNDR in graphene/Si quantum dots-embedded SiO_2_ (SQDs:SiO_2_) multilayers (MLs) tunneling diodes. The LNDR strongly depends on temperature (*T*) as well as on SQD size, and the *T* dependence is consistent with photocurrent (PC)-decay behaviors. With increasing light power, the PC-voltage curves are more structured with peak-to-valley ratios over 2 at room temperature. The physical mechanism of the LNDR, governed by resonant tunneling of charge carriers through the minibands formed across the graphene/SQDs:SiO_2_ MLs and by their nonresonant phonon-assisted tunneling, is discussed based on theoretical considerations.

Si is a principal material in semiconductor industries, but is of limited use in its photonic device applications due to its small- and indirect-bandgap nature. Si quantum dots (SQDs)^1^ that can be tailored based on quantum confinement effect have been employed to get over such intrinsic drawbacks of Si, thereby realizing Si-based optoelectronics. Photonic devices made of SQDs, such as light-emitting diodes (LEDs)^2^,^3^,^4^, solar cells^5^,^6^,^7^,^8^, and photodetectors (PDs)^9^,^10^,^11^, have been fabricated mostly based on a structure of metal/SQDs:SiO_2_ layer/Si wafer/metal by using Al, Au, ITO, Au/Ni, and Au/Sb as metal electrodes. Their device performances, however, are still far below commercial standards. For example, the quantum efficiency, on/off ratio, and responsivity of SQD PDs are currently as low as ∼1% at 530 nm/5 V^9^, ∼3 at ∼9 V^10^, and ∼0.02 A/W at ∼320 nm^11^, respectively. The power efficiencies of SQD LEDs stick around just at 0.1∼0.2%^2^,^3^,^4^ and the energy-conversion efficiencies of SQD solar cells have recently reached 10.4 ∼ 13.0%^5^,^6^,^7^,^8^, much smaller than those of single-crystalline-Si solar cells^12^.

Since the advent of graphene in 2004, its high optical transparency, large carrier mobility, and easy tuning of work function have made it play key roles as transparent electrodes and others in various kinds of graphene-based device structures such as heterostructures with two-dimensional materials^13^,^14^, graphene vertical-tunneling diodes^15^,^16^, graphene-junction Schottky diodes^17^,^18^, and so on. Recently, we have reported graphene/SQDs-embedded SiO_2_ (SQDs:SiO_2_) multilayers (MLs)-heterojunction tunneling diodes^19^ showing high photoresponse that is less-noise, faster, and near-ultra-violet sensitive compared to commercially-available crystalline-Si PDs. Since these results are very promising for significantly enhancing the performances of SQDs-based optoelectronic devices in view of commercial standards, it is highly necessary to carry out in-depth studies on the tunneling-current mechanisms of the graphene/SQDs:SiO_2_ MLs heterojunction diodes.

Negative differential resistance (NDR) has a long history as one of the important tunneling phenomena not only under dark^20^,^21^,^22^,^23^ but also under illumination^24^,^25^,^26^, and has enabled novel applications in a wide range of electronic devices^27^,^28^,^29^,^30^. The NDR behaviors have been also theoretically predicted in graphene^31^,^32^, and experimentally observed in several graphene-based device structures such as heterojunction tunneling transistors^33^, p-n tunneling diodes^15^, field effect transistors^34^,^35^, prompted by the unique two-dimensional properties of graphene at the nanoscale. For the realization of the graphene-based NDR devices, more studies are required to clarify the main mechanism of the NDR, thereby extracting the major controlling factors of the NDR effect in graphene-based device structures. In this work, we report novel features of light-induced NDR (LNDR) first found in graphene/SQDs:SiO_2_ MLs heterojunction tunneling diodes. The LNDR behaviors strongly depend on SQD size (*d*), temperature (*T*), and irradiance power (*P*), and are well explained by resonant tunneling of charge carriers through the miniband formed across the graphene/SQDs:SiO_2_ MLs and by their nonresonant phonon-assisted tunneling. The *T* dependence of the LNDR properties is consistent with that of the lifetimes found in photocurrent (PC)-decay curves. As *P* increases, the photo *I-V* curves are more structured with the peak-to-valley ratios from ∼1.5 to ∼2.2 at room temperature, possibly originating from electric-field screening due to space charge buildup and state filling.

## Results

**Figure 1:**
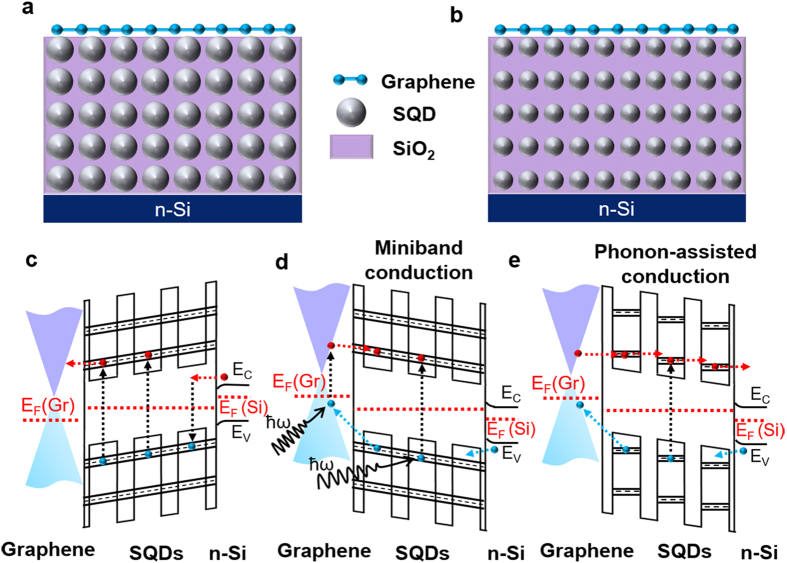
Diagrams of schematic and band structure describing graphene/SQD tunnelling diodes. Schematics of (**a,b**) graphene/SQDs:SiO_2_ MLs heterostructures for typical large and small SQD sizes, respectively. (**c–e**) Band diagrams under forward (miniband conduction) and reverse (miniband and phonon-assisted conductions) biases, respectively, while illuminated. Red and blue spots represent electrons and holes, respectively, contributing to photocurrent. Here, E_F_(Gr) and E_F_(Si) represent the Fermi levels of graphene and Si wafer, respectively.

Figure 1 shows schematic diagrams and band structures of graphene/SQDs:SiO_2_ MLs tunneling diodes, composed of SQDs:SiO_2_ MLs with a total thickness of ∼100 nm, between monolayer graphene sheet and n-type Si wafer (for the fabrication details, see Supplementary Figs S1, S2 and S3). High-resolution transmission electron microscopy (HRTEM) proved regularly-distributed SQDs within SiO_2_ matrix in our previous reports^36^,^37^ (see also Supplementary Fig. S2). Especially, a SiO_2_ layer of ~4 nm thickness, usually located on top of SQDs:SiO_2_ MLs produced by ion beam sputtering deposition and annealing of SiO_2_/SiO_x_ MLs^37^, was etched for better tunneling of charge carriers at the interface of graphene/SQDs:SiO_2_ MLs.

The dark current of the tunneling diodes increases with increasing *d* (Supplementary Fig. S4). In the SQDs:SiO_2_ MLs, the quantum states of the coupled SQDs are overlapped due to the thin (∼2 nm) SiO_2_ barriers, and broaden into minibands (extended Bloch-type states) if the mean free path of the carriers exceeds the ML period, as shown in Fig. 1 (see also Supplementary Fig. S5). Perpendicular transport then proceeds by miniband conduction. However, if the mean free path is smaller than the ML period, the states of the SQDs:SiO_2_ MLs become localized in the SQDs along the direction perpendicular to the layers and conduction proceeds by phonon-assisted tunneling (hopping between adjacent wells)^38^. Under forward bias (*V* > 0 on graphene), the barrier oxide field increases linearly with the applied electric field^2^, resulting in a flow of majority carriers (electrons) through SQDs:SiO_2_ MLs from the substrate (n-Si) to graphene by electron tunneling through minibands, thereby producing a dark current (DC). The rapid increase of current at larger *d* indicates the current is mainly governed by the tunneling probability that is enhanced at larger *d* due to the reduced barrier (SiO_2_) thickness, as shown in Fig. 1a,b. Under reverse bias (*V* < 0) the hole carriers give rise to the main current through phonon-assisted-tunneling conduction (Supplementary Fig. S5). Since the electrons are majority carriers of the n-type substrate and the effective mass of electrons is much smaller than that of holes (which keeps holes much more localized inside SQDs than electrons), much larger current is expected for forward bias than for reverse bias. The estimated diode ideality factor is minimized at *d* = 2.8 nm under forward bias as well as under reverse bias (Supplementary Fig. S4), indicating best-quality tunneling diode at *d* = 2.8 nm.

**Figure 2:**
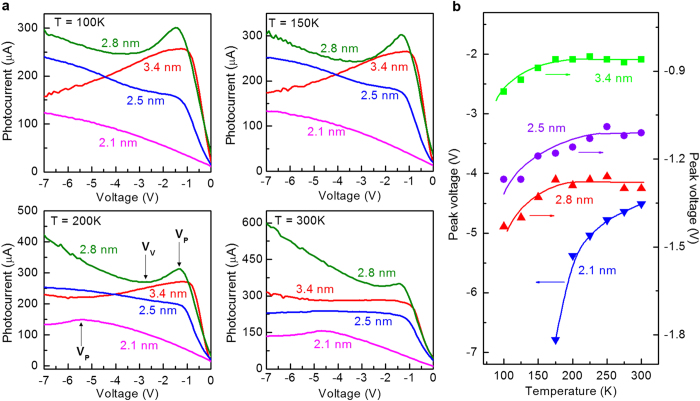
Size- and temperature-dependent photo I-V curves. Size-dependent photo I-V curves of graphene/SQDs:SiO_2_ MLs diodes at various temperatures under reverse bias. The illumination was done with a light power density of 1 mW at a wavelength of 532 nm. As the voltage applied across the RTD terminals is increased from zero, the current increases due to resonant tunneling up to the peak voltage *V*_*P*_. As the voltage across the RTD is increased beyond *V*_*P*_, the current through the diode drops due to reduction in tunneling (non-resonance) until the voltage reaches the valley voltage *V*_*V*_.

Figure 2a shows temperature-dependent PC-vs-voltage (*V*) curves under reverse bias for various SQD sizes. Note that the negligible PC is observed for the forward bias. This can be understood in terms of the slow transit of the electrons in the region of the SQDs:SiO_2_ MLs (see Fig. 1c). Since the holes photoexcited in the SQDs recombine with electrons leaking from the n-type substrate, the PC generated from the photoexcited electrons compensates the loss of net dark current by the recombination, and negligible net PC is therefore observed for the forward bias. However, for reverse bias all photoexcited electrons move to the substrate before the recombination takes place in the tunneling regions (see Fig. 1d). The PC for reverse bias is mostly due to the transport of photogenerated electrons (not holes). The transit time of photoexcited electrons will simply decrease monotonically as the bias voltage increases and therefore the PC increases with the bias voltage. The electrons will not reach the contact if the transit time is smaller than their recombination lifetime.

The most striking feature of the measured PCs in Fig. 2a is the presence of NDR in the PC-*V* curves, i.e., typical behaviors of resonant-tunneling diodes (RTDs)^21^,^22^,^38^. The NDR characteristics depend strongly on *T* as well as *d*. In contrast, such RTD behaviors are not observed in dark *I-V* curves under forward bias as well as under reverse bias, irrespective of *T* and *d* (Supplementary Fig. S6), possibly because the NDR signal is below noise level due to insufficient density of carriers under dark. Typical behaviors of RTD can be described as follows^20^. As the voltage applied across the RTD terminals is increased from zero, the current increases up to the peak voltage *V*_*P*_ due to resonant tunneling, as indicated in Fig. 2a. As the voltage across the RTD is increased beyond *V*_*P*_, the current through the diode drops due to reduction in tunneling (non-resonance) until the voltage reaches the valley voltage *V*_*V*_, as indicated in Fig. 2a. Beyond *V*_*V*_, the current through the device increases again as in conventional diodes. The *T* dependence of the peak voltages for each *d* are summarized in Fig. 2b.

**Figure 3:**
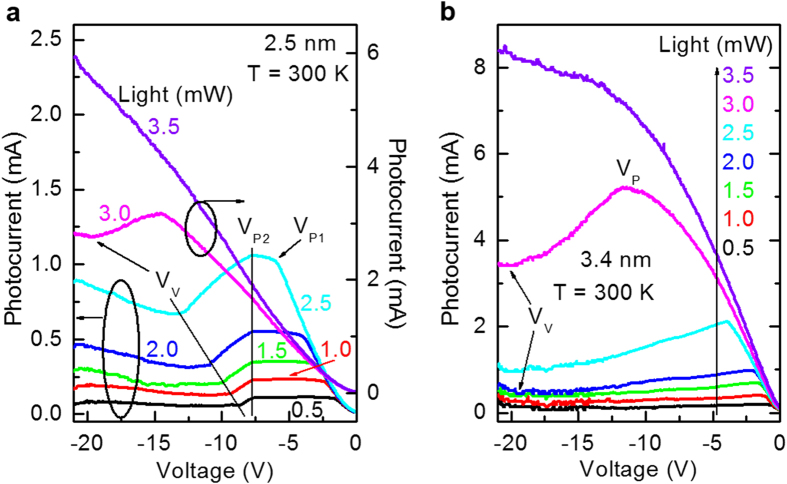
Light-power-dependent photo I-V curves (**a,b**) Evolution of photo *I*-*V* curves of graphene/SQDs:SiO_2_ MLs diodes at room temperature for *d* = 2.5 and 3.4 nm, respectively under various light power intensities from 0.5 to 3.5 mW. 1^st^ peak voltage *V*_*P1*_, 2^nd^ peak voltage *V*_*P2*_, and *V*_*V*_ for *d* = 2.5 nm, and *V*_*P*_ and *V*_*V*_for *d* = 3.4 nm are indicated.

**Figure 4:**
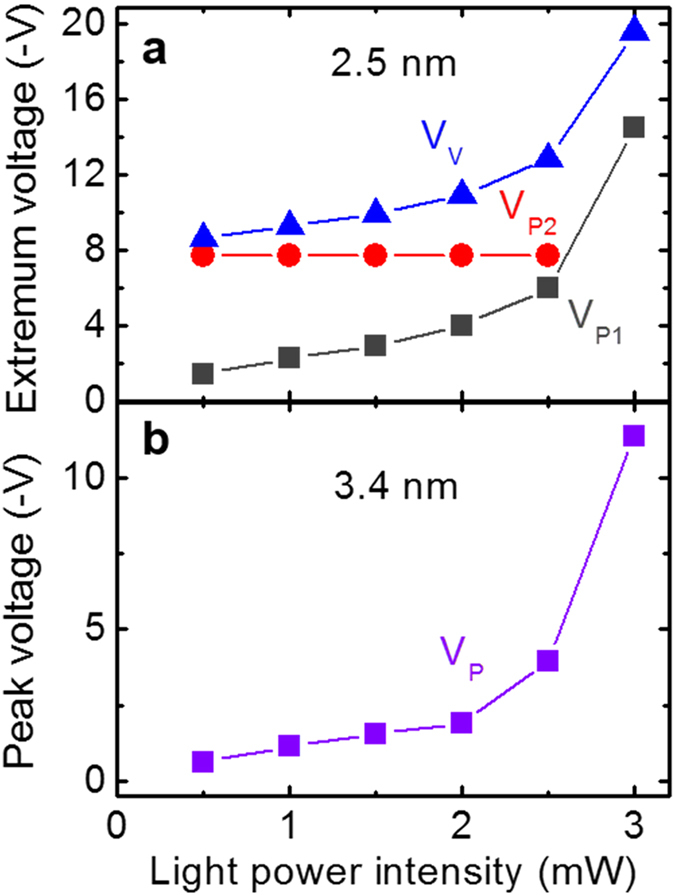
LNDR parameters (**a**) Temperature-dependent variation of the peak voltages obtained in the photo *I*-*V* curves of graphene/SQDs:SiO_2_ MLs diodes for four different SQD sizes. (**b**) Room-temperature 1^st^- and 2^nd^-peak and valley voltages as functions of light power density for *d* = 2.5 nm. (**c**) Room-temperature peak voltage as a function of light power density for *d* = 3.4 nm.

The NDR behaviors seem to strongly depend on the density of charge carriers, judging from no finding of the phenomenon without illumination. And there have been several reports on the dependence of the NDR behaviors on the carrier density in III-V superlattices^25^,^38^. By similar experiments for various light powers in a wider range of reverse bias down to −20 V, the photo *I-V* curves more structured with peaks and valley were obtained for *d* = 2.5 nm, as shown in Fig. 3a, but no such big changes were observed for other three kinds of diodes except the *P*-dependent monotonic shift of *V*_*P*_, as shown for *d* = 3.4 nm in Fig. 3b. To analyze the NDR behaviors for *d* = 2.5 nm, the 1^st^ peak voltage *V*_*P1*_, the 2^nd^ peak voltage *V*_*P2*_, and *V*_*V*_ are indicated in Fig. 3a, and their *P*-dependences are summarized in Fig. 4a.

**Figure 5:**
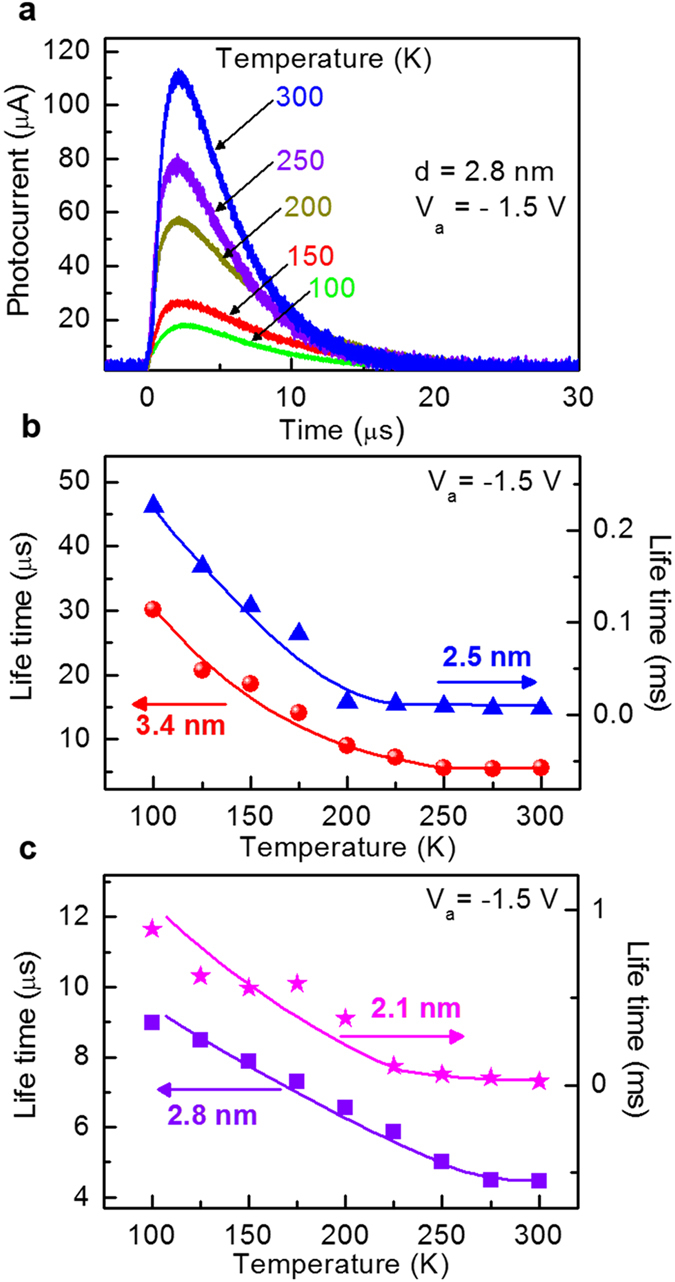
Transient photocurrent and lifetimes. (**a**) Photocurrent decay curves at various temperatures from 77 to 300 K for *d* = 2.8 nm. (**b,c**) Lifetimes as functions of temperature obtained from the photocurrent decay curves for *d* = 2.1, 2.5, 2.8, and 3.4 nm. The bias voltage (*V*_*a*_) of −1.5 V was applied for these photocurrent decay experiments.

To probe the charge-transfer dynamics, transient PCs were recorded for various bias voltages and temperatures, as shown in Fig. 5a. The turn-on transient response is characterized by a relatively fast increase in the PC during several tens of ns, followed by a relatively slow decay to a steady state value during several μs ∼ ms, which is attributed to electron–hole recombination.

## Discussion

The observed NDR phenomena can be understood by considering the miniband formation in the SQDs:SiO_2_ MLs structure, as explained above. Since at the valley voltage *V*_*V*_ the electron wave functions become strongly localized within each SQD region, the overlapping of the wave functions decreases and the current is therefore reduced, which results in NDR. The threshold condition for the decrease in the overlapping of the wave functions is given approximately by e*V*_*d*_ ~ Δ*E*. Here, *V*_*d*_ is the voltage drop across the ML period (i.e., the voltage drop between two electrodes becomes *V*_*d*_*N*, where *N* is the number of MLs) and Δ*E* is the width of the miniband. Thus, the peak voltage *V*_*P*_ ≈ Δ*EN/e*. As a result, the NDR in the SQDs:SiO_2_ MLs occurs when the potential energy difference between two adjacent SQDs in the MLs exceeds Δ*E*, which corresponds to the transition from band-like conduction to phonon-assisted-tunneling (hopping) conduction between the localized states of the SQDs. Clearly, this gives rise to NDR because the spatial overlap between the states of neighboring QDs decreases with increasing bias voltage. For example, no NDR is observed at temperatures below 200 K for the smallest SQD system (*d* = 2.1 nm), as shown in Fig. 2a. Below 200 K the PC increases monotonically with increasing reverse bias. However, above 200 K the PC-*V* curves show well-defined NDR behaviors. This phenomenon can be explained by the decrease of the recombination lifetime in the non-resonant tunneling regions at high temperatures.

The energy dispersion relation of the miniband is given by the simple one-dimensional model as *E*(*k*) = *E*_*0*_ [1 − cos(*kd*_*s*_)], where 2*E*_*0*_ (=Δ*E*) is the band width and *d*_*s*_ is the average distance between SQD layers (i.e., ML period). *E*_*0*_ (=Δ*E/2*) is proportional to the tunneling probability (*T*_*P*_) through the barrier which can be approximated by *T*_*P*_ ≈ exp 
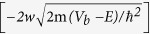
, where *V*_*b*_ is the barrier height and *w* is the width of the potential barrier. For larger *w*, *T*_*P*_ is exponentially suppressed, as described in Fig. 1, and so the band width Δ*E* becomes smaller. As summarized in Fig. 2b, the *V*_*P*_ increases with increasing temperature *T* for the four different SQD sizes. The current increases with increasing the bias voltage up to *V*_*P*_ by the miniband conduction, as shown in Fig. 1, but by further increase of the bias from *V*_*P*_ to *V*_*V*_, the current decreases through the phonon-assisted tunneling. The resonance through the miniband occurs again at a higher bias voltage, resulting in a current increase above *V*_*V*_, consistent with the results in Fig. 2a. As *T* is increased, the phonon scattering becomes stronger due to larger population of optical phonons at higher *T*. The phonon-assisted tunneling gives rise to the shift of *V*_*P*_, which is more sensitive to *T* at smaller *d*, as shown in Fig. 2b, because the width of miniband is reduced at smaller *d*. The PC decay time or the life time decreases with increasing the temperature due to the enhanced phonon-assisted tunneling, as shown in Fig. 5b,c (see also Supplementary Fig. S7), consistent with the *T*-dependent variations of the peak voltages, as shown in Fig. 2b.

As shown in Fig. 3a, with increasing *P*, the plateau between *V*_*P1*_ and *V*_*P2*_ shrinks, and finally, it disappears as the two peaks merges at *P* = 3.0 mW. In contrast, the slope between *V*_*P2*_ and *V*_*V*_ expands as *P* increases. The peak (*V*_*P2*_)-to-valley (*V*_*V*_) ratio varies from 1.57 to 1.84 in the range of *P* between 0.5 and 2.5 mW. These evolutions suggest that a blocking process in carrier transport exists around extrema *V*_*P1*_, *V*_*P2*_, and *V*_*V*_ under high carrier densities. The shape of the photo *I*-*V* curve as a function of *P* is thought to be strongly affected by electric-field screening due to space charge buildup and state filling^25^,^38^. If we assume that each carrier transport path has a limit in the capacity to allow the flow of carriers, the current will saturate at high carrier density. The blocked carriers exceeding the flow capacity will induce an electric field screening that can alter the internal electric field in the SQD MLs. As shown in Figs 3a and 4a, current peak *V*_*P1*_corresponding to the resonant tunneling through the miniband moves toward a higher voltage as the excited carrier density is increased. Simultaneously, valley *V*_*V*_, which is thought to correspond to increasing nonresonant phonon-assisted tunneling, also moves towards higher bias voltage. These behaviors can be explained by the electric-field screening generated by the space charges. Even when the carrier transport may be dominated by the resonant-tunneling-transfer path, it is considered that the carriers cannot enter into the miniband states in adjacent SQD filled by the optical excitation at point *V*_*P1*_. This will saturate the current up to *V*_*P2*_. Note that the position of *V*_*P2*_ is almost invariant. This means that the state filling is reached faster when the carrier density is higher, thereby causing such a pinning of *V*_*P2*_ for various light powers. The *P*-dependent monotonic shift of *V*_*P*_ for *d* = 3.4 nm, as shown in Figs 3b and 4b, can be similarly attributed to the electric-field screening. For this sample, the peak (*V*_*P*_)-to-valley (*V*_*V*_) ratio varies from 1.53 to 2.21 in the range of *P* between 0.5 and 3.0 mW.

In these tunneling diodes we use graphene as an electrode. In addition to the electronic/optical advantages of graphene, graphene as a transparent electrode plays an important role in the observation of NDR in photo I-V curves. Unlike the devices with metal electrodes, the tunneling diodes with graphene electrodes give rise to the NDR behaviors in the wide range of light wavelength if the photon energy is greater than the band gap of SQDs. We also mention that the NDR behaviors can be adjusted by tuning the Fermi energy of graphene, which can be achieved by doping (or gating). The density of states of graphene affects the peak to valley ratio because the density of states of graphene is zero (or minimum) at the Dirac point. The shift of Dirac point with varying the applied bias gives a strong effect on the observed current because the current directly depends on the density of states.

The achievements in this work (e.g., the large ratio of the peak to valley current at room temperature) will enhance the possibilities that the heterojunction diodes can be employed in optoelectronic devices such as optical modulators, optical switches, optical memories, and photodetectors for imaging, sensing, recording, and communications.

## Methods

### Device Fabrication

SiO_x_/SiO_2_ MLs with 50 periods of 2 nm thin layers were grown on n-type (100) Si wafers at room temperature (RT) using an Ar^+^ beam with an ion energy of 750 eV and a Si target under oxygen atmosphere in a reactive ion beam sputtering system with a Kaufman type DC ion gun (Supplementary Fig. S1a). Details of the system are described elsewhere^39^. The deposition chamber was evacuated to a pressure of 5.0 × 10^−9^ Torr before introducing argon gas into the system. The stoichiometry of the SiO_x_ films could be analyzed and controlled with *in-situ* x-ray photoelectron spectroscopy using Al *kα* line of 1486.6 eV. After deposition, the SiO_x_/SiO_2_ MLs were annealed at 1100 ^o^C in a ultra-pure nitrogen ambient by using a horizontal furnace to form SQDs MLs regularly embedded in SiO_2_ (SQDs:SiO_2_ MLs). HRTEM proved regularly-distributed SQDs within SiO_2_ matrix (Supplementary Fig. S2a). For the *x* values of 0.8, 1.0, 1.2, and 1.4, the average sizes of SQDs were estimated to be 3.4, 2.8, 2.5, and 2.1 nm (Supplementary Fig. S2b), corresponding to the photoluminescence (PL) wavelengths of 772, 762, 725, and 716 nm, (1.61, 1.63, 1.71, and 1.73 eV) respectively^40^. The absorption peak energies are 1.81, 1.93, 2.14, and 2.20 eV, respectively, obtained from the PC spectra for the four samples. In contrast, the SQD density is (3.2∼3.6) × 10^12^ cm^−3^ without a big variation over the same *x* range. Graphene layers were grown on 70-μm-thick Cu foils (Wacopa, 99.8 purity) in a graphite-heater-based chemical-vapor-deposition quartz tube furnace at a growth temperature of 1000 ^o^C with 10-sccm H_2_ and 20-sccm CH_4_ flowing at a pressure of 3 Torr^41^,^42^. The graphene/Cu stack was spin-coated with poly(methyl methacrylate) (PMMA), and the Cu was then etched in a 1 M ammonium persulfate for 10 h. The graphene/PMMA stack was then placed in deionized water before transferring to the 100 nm SQDs:SiO_2_ MLs/n-type Si wafers and blow-dried with dry N_2_. The PMMA/graphene/SQDs:SiO_2_ MLs/n-Si stack was then heated on a hot plate in air at 180 ^o^C for 2 h to cure the PMMA. After the samples were cooled to RT, the PMMA was stripped by soaking them in acetone for 1 h at RT. Subsequently, the graphene/SQDs:SiO_2_ MLs/n-Si stack was put in isopropyl alcohol for 10 min and dried by blowing N_2_ to minimize the water traps possibly present at the graphene/SQDs:SiO_2_ MLs interface, and annealed at 400 ^o^C for 1 h in vacuum to remove the surface adsorbates^42^. After the transfer, the graphene layers were characterized by atomic force microscopy (AFM), Raman spectroscopy, and PL. The root-mean-square roughness was estimated to be ~0.26 nm and ~1.4 nm on the SQDs:SiO_2_ MLs and the graphene/SQDs:SiO_2_ MLs, respectively by AFM height profiles (Supplementary Fig. S3a). The AFM image and height profile (Supplementary Fig. S3b) proved that the graphene is single layer. The Raman intensity ratio (I_G_/I_2D_) of the G and 2D bands peaked at ~1585 and ~2683 cm^−1^, respectively, was about 0.47 on the graphene/SQDs:SiO_2_ MLs (Supplementary Fig. S3c) and the transmittance at 500 nm was 97.7% (Supplementary Fig. S3d), indicative of single-layer graphene. The PL intensity and decay time of SQDs after the graphene transfer were almost invariant (Supplementary Fig. S3e,f), meaning nearly-perfect transmittance of single-layer graphene. For the electrical contacts of 5 × 5 mm^2^ size, Cr and Au films were successively deposited on the top of graphene as well as on the bottom of n-Si substrate by using a shadow mask in an electron beam evaporation system, thereby completing the graphene/SQDs:SiO_2_ MLs heterojunction diodes, as shown in Fig. 1a.

### Device Characterization

*I-V* measurements to characterize the electrical behaviors of the diodes were carried out under dark and illumination by using a Keithley 2400 source meter controlled by a LabView program (for the experimental setup, see Supplementary Fig. S1b). The illumination was done by a 532-nm diode laser at light powers of 0.5∼3.5 mW. During the measurements, the diodes were mounted in a dark, electrically-shielded, and optically-sealed chamber under vacuum on the optical table to reduce vibrational noise. Transient photocurrent studies were conducted using laser pulses from the Nd:YAG laser. (532-nm wavelength, 20-ps duration, and 20-Hz repetition rate, generated by a Continuum Leopard-D20 Nd:YAG laser), which were focused onto the devices with a spot size of ~5 × 5 mm^2^. A laser power meter (Laser probe, Rj-760) was used to measure the average power of the laser pulses. The laser pulse temporal profiles and transient PC were monitored with the 50-ohm-terminated, 500-MHz–bandwidth digital oscilloscope (Tektronix DPO 4054)^41^.

## Additional Information

**How to cite this article**: Lee, K. W. *et al*. Light-induced negative differential resistance in graphene/Si-quantum-dot tunneling diodes. *Sci. Rep.* **6**, 30669; doi: 10.1038/srep30669 (2016).

## Supplementary Material

Supplementary Information
