# Supplementary material for: Light-induced negative differential resistance in graphene/Si-quantum-dot tunneling diodes
Source: Sci Rep. 2016 Jul 28;6:30669. doi: 10.1038/srep30669 (PMC4964343; doi:10.1038/srep30669)
Supplement: Supplementary Information [file srep30669-s1.doc]

**Supplementary Information for**

Light-induced negative differential resistance in graphene/Si-quantum-dot tunneling diodes

Kyeong Won Lee,^1^*Chan Wook Jang,^1^* Dong Hee Shin,^1^Jong Min Kim,^1^ Soo Seok Kang,^1^ Dae Hun Lee,^1^ Sung Kim,^1^ Suk-Ho Choi,^1†^ Euyheon Hwang^2^

^1^Department of Applied Physics and Institute of Natural Scineces, Kyung Hee University, Yongin 446-701, Korea

^2^SKKU Advanced Institute of Nanotechnology, Sungkyunkwan University, Suwon 440-746, Korea

^*^These authors contributed equally to this work.

^†^Corresponding author: [sukho@khu.ac.kr](mailto:sukho@khu.ac.kr)

**Supplementary Figures**

**Fig. S1.** (a) Summary of the procedures for fabricating the graphene/SQDs:SiO_2_ MLs devices. (b) Experimental setup.

**Fig. S2.** (a) Cross-sectional high-resolution TEM image of 50-period 2 nm SiO_1_*.*_0_/2 nm SiO_2_ MLs after annealing at 1100 ^o^C. The inset shows a magnified TEM image of a single SQD. The scale bar in the inset is 2 nm. (b) Average SQD size as a function of x value.

**Fig. S3.** (a) AFM images and height profiles of SQDs:SiO_2_ MLs and graphene/SQDs:SiO_2_ MLs on n-Si wafer. (b) AFM image and height profile of graphene/SQDs:SiO_2_ MLs step. (c) Raman spectrum of pristine graphene. The intensity ratios of D to G and G to 2D Raman bands are indicated. (d) Transmittance of single-layer graphene (e) PL spectra of SQDs:SiO_2_ MLs and graphene/ SQDs:SiO_2_ MLs on n-Si wafer. (f) PL decay curves of SQDs:SiO_2_ MLs and graphene/ SQDs:SiO_2_ MLs on n-Si wafer. Here, GR indicates graphene.

**Fig. S4.** Dark *I-V* curves for various SQD sizes. The inset shows Ideality factors as functions of SQD size under forward and reverse biases.


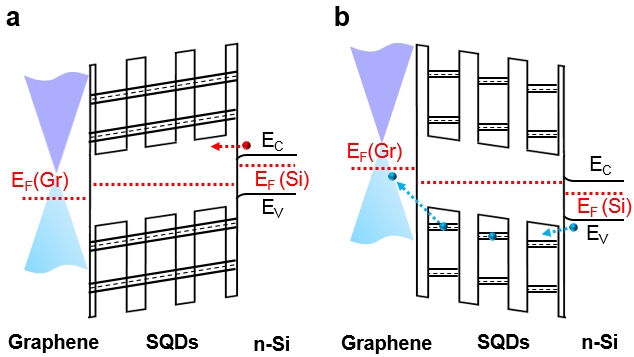


**Fig. S5.** Band diagrams under (a) forward (miniband conduction) and (b) reverse (phonon-assisted conduction) biases.

**Fig. S**6. Temperature-dependent dark I-V curves for various SQD sizes.

**Fig. S7.** Temperature-dependent lifetimes at various reverse biases for d = 2.8 nm.
